# Case Report: Intracardiac echocardiography-guided pulmonary vein stenting for pulmonary vein stenosis caused by a left atrial tumor

**DOI:** 10.3389/fcvm.2026.1927233

**Published:** 2026-09-08

**Authors:** Yoonmin Shin, Hoyoung Son, Namsik Yoon, Juhan Kim

**Affiliations:** 1 Department of Cardiology, Mokpo Hankook Hospital, Mokpo, Republic of Korea; 2 Department of Cardiology, Chonnam National University Hospital, Gwangju, Republic of Korea; 3 Department of Internal Medicine, Chonnam National University Medical School, Gwangju, Republic of Korea

**Keywords:** angiosarcoma, intracardiac echocardiography, left atrium, pulmonary vein stenosis, stenting

## Abstract

**Background:**

Pulmonary vein stenosis (PVS) caused by a cardiac tumor is rare, and experience with transcatheter pulmonary vein stenting is limited. We report a challenging case of symptomatic PVS managed with intracardiac echocardiography (ICE)–guided pulmonary vein stenting as palliative treatment.

**Case:**

An 83-year-old woman presented with progressive dyspnea for 4 months. Electrocardiography, chest x-ray, and routine laboratory tests were unremarkable. Transthoracic echocardiography revealed a large left atrial mass involving the right pulmonary vein and causing stenosis. Multimodality imaging, including cardiac computed tomography (CT), cardiac magnetic resonance imaging, and ^1^⁸F-FDG PET/CT, suggested primary cardiac angiosarcoma. Considering the patient's advanced age and poor surgical candidacy, we selected a palliative transcatheter intervention. Under ICE guidance, transseptal puncture was performed. Pulmonary venography revealed severe stenosis of the right inferior pulmonary vein. After sequential balloon angioplasty, a 14 × 40 mm self-expanding stent was successfully implanted with adjunctive balloon dilation. Final angiography demonstrated complete restoration of pulmonary venous flow without residual stenosis or procedural complications. The patient's dyspnea improved markedly, and she was discharged 2 days after the procedure.

**Conclusion:**

In selected patients with PVS who are unsuitable for surgical treatment, ICE-guided pulmonary vein stenting can be performed safely and may provide effective palliation by restoring pulmonary venous flow and relieving symptoms.

## Introduction

Pulmonary vein stenosis (PVS) is an uncommon condition that most often occurs as a complication of pulmonary vein ostial ablation for atrial fibrillation or, less commonly, as a congenital abnormality. Malignancy-related PVS is rare, particularly when caused by a primary cardiac angiosarcoma. Cardiac angiosarcoma is the most common primary malignant cardiac tumor, but its clinical presentation is often nonspecific, making early diagnosis challenging. Progressive pulmonary vein obstruction may result in pulmonary venous hypertension, dyspnea, recurrent pulmonary edema, and impaired quality of life. Surgical resection remains the preferred treatment whenever feasible; however, many patients present with advanced disease or are poor surgical candidates because of tumor extent or advanced age. In such circumstances, palliative interventions aimed at relieving pulmonary venous obstruction may improve symptoms. However, experience with transcatheter pulmonary vein stenting for PVS caused by cardiac angiosarcoma is limited. We report a challenging case of intracardiac echocardiography (ICE)-guided pulmonary vein stenting performed for symptom relief in an older adult with suspected primary cardiac angiosarcoma.

## Case description

**Figure 1:**
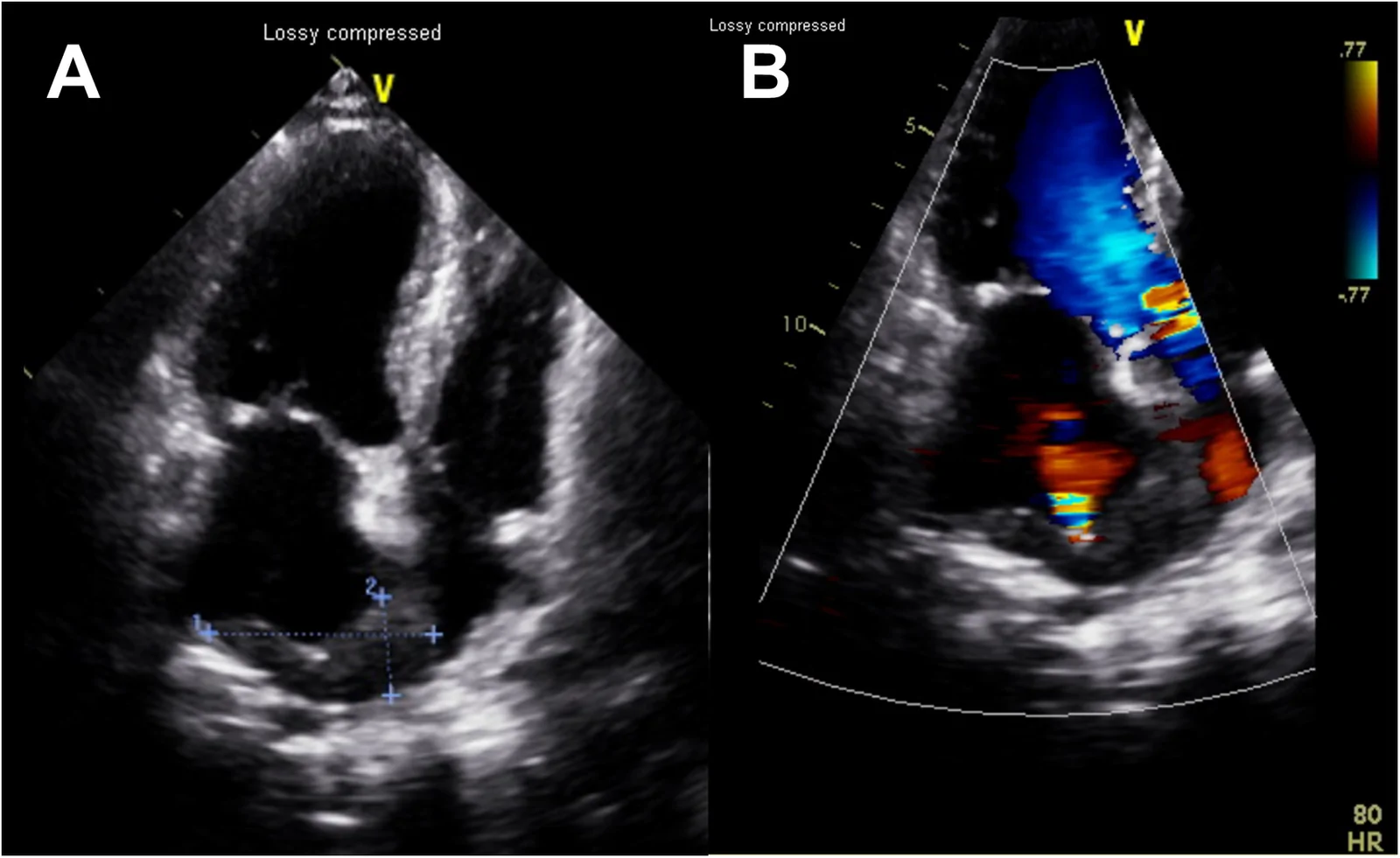
**(A)** Transthoracic echocardiography apical four chamber view showing broad mass (22.4 × 51.0 mm) with moderate echogenicity at posterior wall of the left atrium. **(B)** Color Doppler demonstrating an inflow jet from right pulmonary vein.

**Figure 2:**
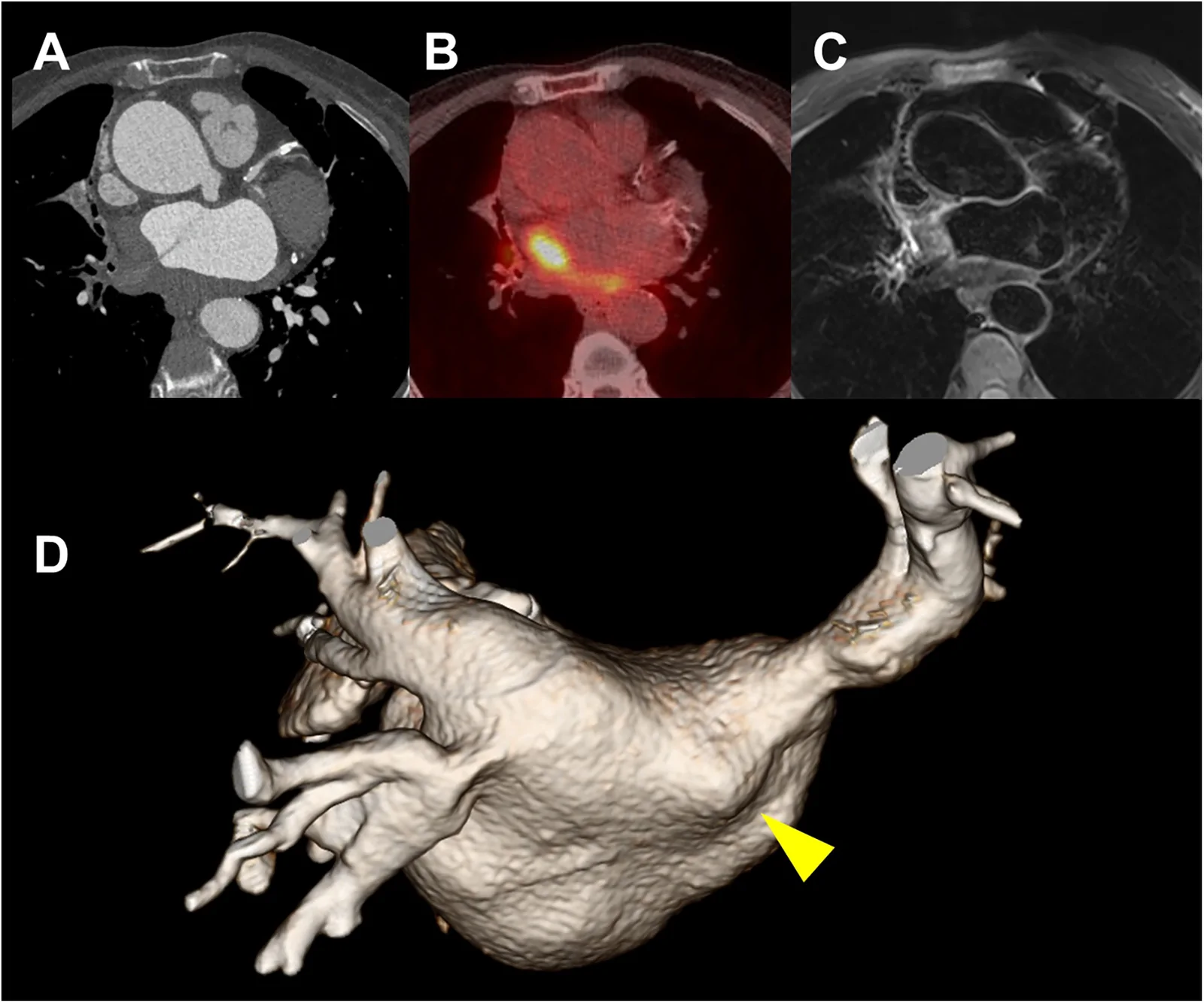
**(A)** and **(D)** cardiac computed tomography demonstrated the left atrial wall mass and obliteration (yellow arrow head) of right inferior pulmonary vein. **(B)** Torso PET-CT(F-18 FDG) showed heterogeneous hypermetabolic lesion in the left atrium posterior wall. **(C)** Axial black-blood T2-weighted magnetic resonance image showing a heterogeneous hyperintense left atrial mass, suggestive of a malignant cardiac tumor.

**Figure 3:**
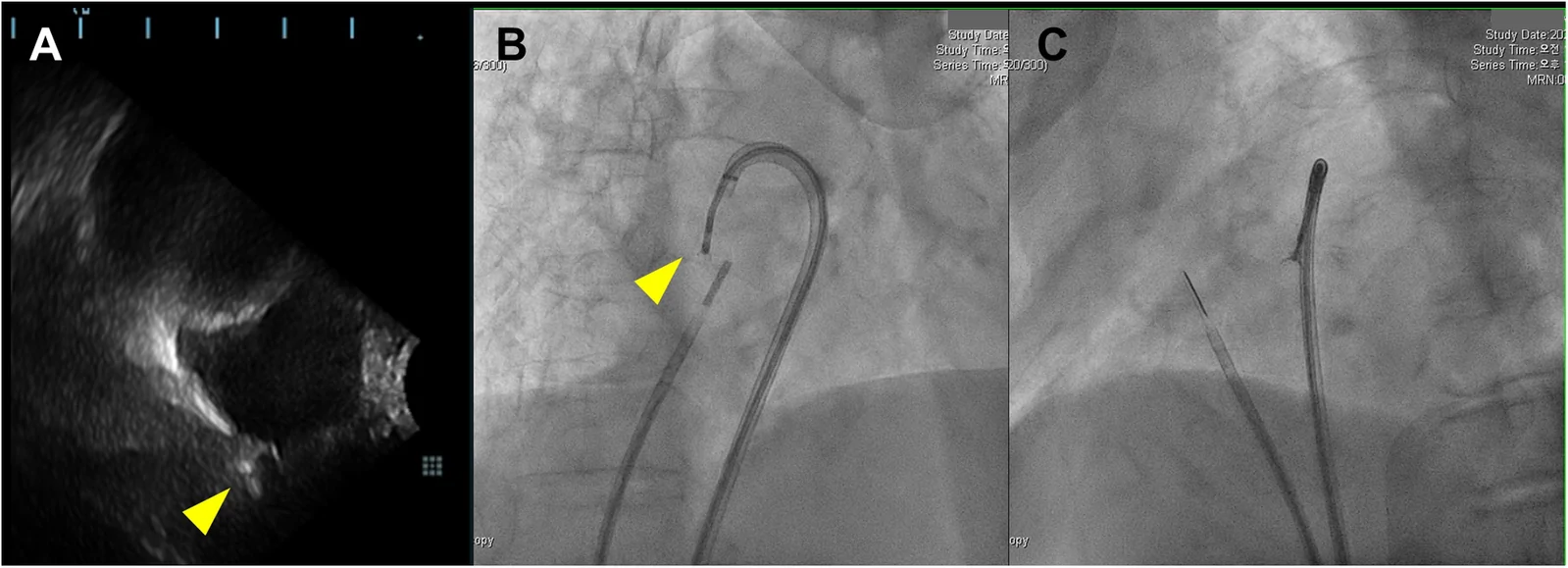
Intracardiac echocardiography guided left atrium forcep biopsy. **(A)** The biopsy forcep is visible (yellow arrow head). **(B)** and **(C)** The fluoroscopic right anterior oblique and left anterior oblique image during the biopsy using a deflectable sheath.

**Figure 4:**
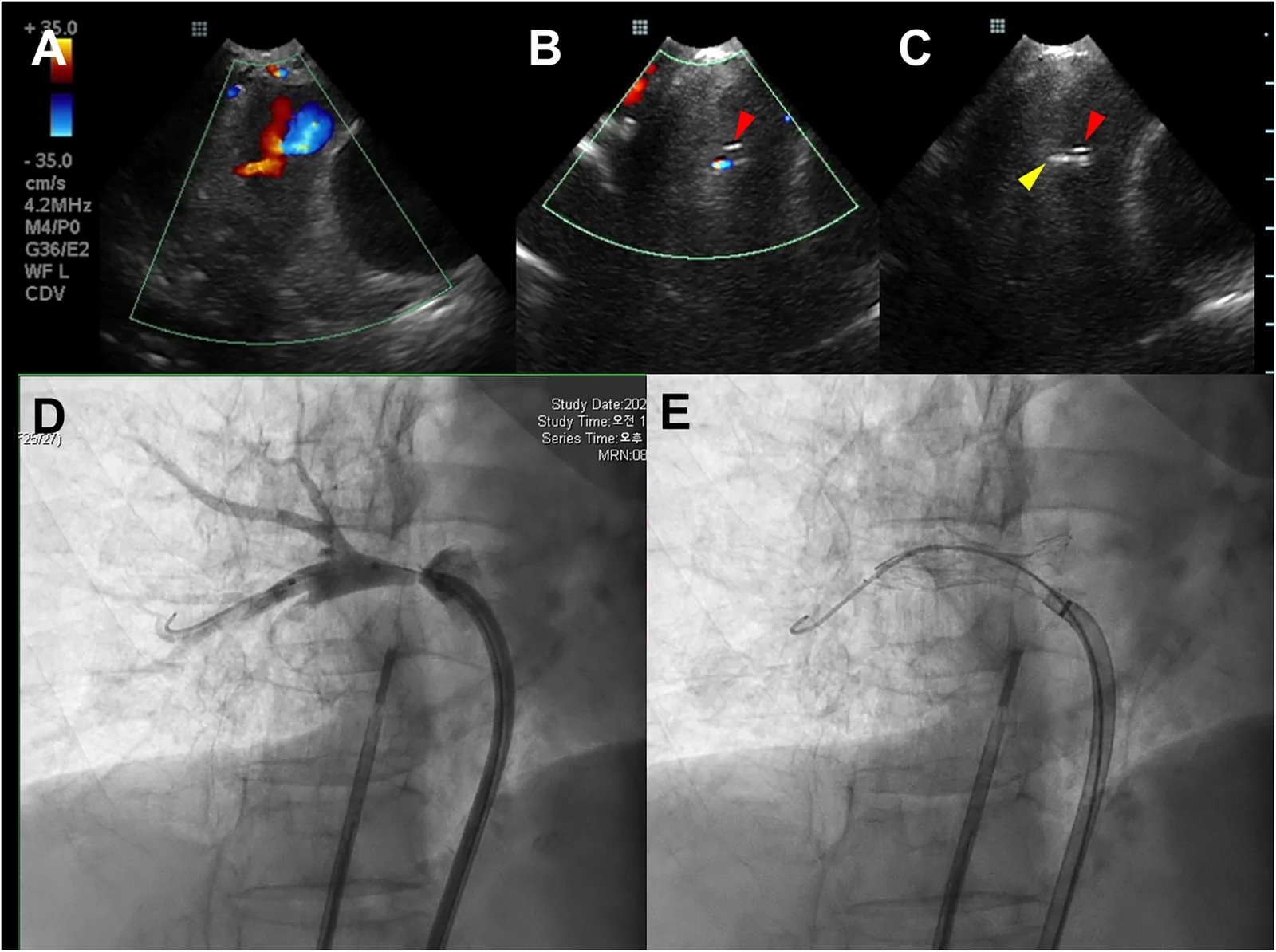
Intracardiac echocardiography guided wiring and stenting in right inferior pulmonary vein. **(A)** Intracardiac echocardiography showing inflow jet from the right inferior pulmonary vein. **(B)** The guiding sheath is deflected tracking the small inflow jet color. The sheath tip (red arrow head) and inflow jet are visible. **(C)** A Radifocus® guidewire (Terumo, Tokyo, Japan) (yellow arrow head) is engaged into the right inferior pulmonary vein ostium through the sheath (red arrow head). **(D)** and **(E)** Angiogram showed critical stenosis at the right inferior pulmonary vein ostium. After balloon angioplasty, a 14 × 40 mm Protégé™ GPS self-expanding peripheral stent was implanted with post adjunctive balloon dilatation.

An 83-year-old woman presented to our heart center with dyspnea that had progressed over 4 months. Electrocardiography, chest x-ray, and routine laboratory studies, including cardiac enzymes, did not reveal a specific cause of dyspnea. Transthoracic echocardiography detected a large mass (22.4 × 51.0 mm) attached to the left atrium along the right inferior pulmonary vein (RIPV) (Figure 1A). Color Doppler suggested PVS (Figure 1B). Differentiation from organized thrombus was required. For better characterization of the mass, we performed cardiac computed tomography (CT), ^1^⁸F-FDG PET/CT, and cardiac magnetic resonance imaging (MRI). Cardiac CT demonstrated a left atrial posterior wall mass that was encasing the RIPV with near-complete obliteration (Figures 2A,D). ^1^⁸F-FDG PET/CT showed heterogeneous hypermetabolic uptake in the left atrial posterior wall (Figure 2B). Axial black-blood T2-weighted cardiac MRI showed a heterogeneous hyperintense left atrial mass (Figure 2C). These findings were suggestive of a malignant cardiac tumor, but biopsy was required to confirm the diagnosis and guide further treatment. Given the patient's functional status and advanced age, we selected palliative intervention rather than definitive surgery. After transseptal puncture, ICE (8-Fr AcuNav™ catheter, Johnson & Johnson MedTech, Irvine, CA, USA) guided left atrial mass biopsy was performed (Figure 3). ICE showed an inflow jet from the RIPV (Figure 4A). The guiding sheath was engaged into the RIPV ostium by tracking the small inflow jet on color Doppler (Figure 4B). Pulmonary vein ostial angiography showed severe stenosis in the RIPV ostium (Figure 4D). With the aid of ICE, a Radifocus® guidewire (Terumo, Tokyo, Japan) was advanced across the RIPV ostial stenosis (Figure 4C). Figures 4A, B, and C were obtained with the ICE catheter positioned in the right atrium. From the standard home view, clockwise rotation of the ICE catheter sequentially visualized the left pulmonary veins, the RIPV, and the right superior pulmonary vein. Because the ICE catheter was located close to the RIPV and the ultrasound beam intersected the long axis of the RIPV at an oblique angle, only the pulmonary vein ostium was visualized on ICE. However, color Doppler imaging allowed visualization of the residual pulmonary venous inflow as a jet entering through the pulmonary vein ostium (Figures 4A,B), which was useful for identifying the stenotic orifice and confirming guidewire passage across the lesion. A 5-Fr multipurpose catheter was engaged into the RIPV, and contrast injection confirmed its location. Stepwise balloon dilatation was performed using 4.0 × 20 mm and 9.0 × 20 mm Admiral™ PTA balloon catheters (Medtronic, Minneapolis, MN, USA). After balloon angioplasty, a 14 × 40 mm Protégé™ GPS self-expanding peripheral stent (Medtronic, Minneapolis, MN, USA) was implanted in the RIPV with adjunctive post-dilation using a 9.0 × 20 mm Admiral™ PTA balloon catheter (Medtronic, Minneapolis, MN, USA) (Figure 4E). After stent implantation, the peak pulmonary vein inflow velocity decreased from 1.7 to 1.4 m/s, corresponding to an estimated reduction in the pressure gradient of approximately 4 mmHg. The inflow from the RIPV was restored after stent implantation. There was no residual stenosis, dissection, or pericardial effusion after the procedure. The patient's dyspnea improved markedly, and she was discharged 2 days after the procedure. Unfortunately, the biopsy was non-diagnostic. At the 3-month follow-up, the peak pulmonary vein inflow velocity was 1.17 m/s, and the patient did not complain of dyspnea. Palliative management for the underlying malignancy was continued.

## Discussion

PVS is an uncommon clinical condition that is most frequently associated with congenital abnormalities or acquired injury following pulmonary vein isolation procedures for atrial fibrillation. Malignancy-related PVS is exceedingly rare, particularly when caused by primary cardiac angiosarcoma, and reports describing pulmonary vein stenting in this setting remain extremely limited (1).

The diagnosis of cardiac angiosarcoma is challenging because symptoms are often nonspecific and conventional tests may be unrevealing. In this case, the patient presented with progressive dyspnea, yet electrocardiography, chest radiography, and laboratory studies provided limited diagnostic insight. Multimodality imaging, including transthoracic and transesophageal echocardiography, cardiac CT, cardiac MRI, and ^1^⁸F-FDG PET/CT, was essential for identifying the left atrial mass and suggesting a malignant etiology. Nevertheless, histopathological confirmation remained necessary to establish the definitive diagnosis and guide the treatment strategy (1, 2). However, biopsy carries potential risks, including bleeding, embolization, and cardiac perforation. In patients with advanced disease, the decision to perform biopsy should balance these procedural risks against the potential benefit of establishing a definitive diagnosis to guide palliative treatment and multidisciplinary management (1). Despite accepting these procedural risks to establish a definitive diagnosis, the biopsy specimen obtained in the present case was ultimately non-diagnostic.

This case also illustrates the value of ICE during complex structural interventions. ICE can provide real-time guidance for transseptal puncture, tissue biopsy, guidewire manipulation across the stenotic pulmonary vein, and surveillance for procedural complications (1, 2). This enables accurate guidewire crossing, confirms restoration of pulmonary venous inflow immediately after stent deployment, and allows continuous monitoring for procedural complications without additional contrast injection. In the present case, ICE contributed substantially to procedural safety and technical success. Although ICE provides excellent real-time intraprocedural guidance, its field of view is smaller than that of transesophageal echocardiography, and visualization of the distal pulmonary vein may be limited. Successful ICE-guided intervention requires familiarity with standardized imaging views, careful catheter manipulation from the home view, and recognition of key anatomical landmarks to obtain optimal imaging planes and maintain consistent procedural orientation (3).

Malignant PVS is associated with poor prognosis because it usually reflects advanced disease. Medical treatment, including chemotherapy, radiotherapy, and anticoagulation, may reduce tumor burden and relieve symptoms in selected patients; however, improvement is often limited and pulmonary venous obstruction frequently persists. Pulmonary vein stenting may represent a useful palliative treatment option in selected patients considering tumor stage, life expectancy, symptom severity, and procedural risk (4–6). In our patient, severe stenosis of the RIPV resulted in symptomatic pulmonary venous obstruction. Because balloon angioplasty alone is associated with a substantial risk of restenosis, stent implantation was performed to achieve a larger luminal diameter and potentially improve long-term vessel patency. Following stent deployment, pulmonary venous flow was restored successfully without procedural complications, leading to significant symptomatic improvement and early hospital discharge.

However, this case also provides several important clinical lessons for patients with cardiac angiosarcoma who are not candidates for definitive treatment. Careful assessment of the potential benefits and risks of intervention is essential. First, if the obstructive lesion contains a substantial thrombotic component, the risk of thromboembolic complications may be considerable. Second, symptom relief may not be achieved when only a single pulmonary vein stenosis is treated, particularly in patients with multifocal pulmonary venous involvement. Third, the biological characteristics of the tumor and the morphology of the stenotic channel may affect procedural feasibility and durability. Therefore, the role of pulmonary vein intervention in this patient population remains uncertain and should be individualized. Additional case experience and longer-term follow-up are needed to define safety, efficacy, and clinical utility of this approach (1, 5, 6). Intracardiac tumor-associated thrombosis is difficult to diagnose because clinical manifestations are often nonspecific. Multimodality imaging is essential for differentiating malignant tumors from thrombus and assessing their anatomical extent. Management should be individualized according to tumor burden, cardiac involvement, and overall prognosis (7). In the present case, neither the size of the left atrial mass nor the pulmonary venous inflow changed after 3 weeks of anticoagulation therapy. Furthermore, cardiac MRI did not demonstrate imaging features suggestive of thrombus, making a friable thrombus less likely. Fortunately, the mass did not involve the other pulmonary veins except the RIPV, allowing restoration of pulmonary venous drainage with a single stent. The patient experienced immediate and marked improvement in dyspnea following the procedure. Because the biopsy and intervention were performed during the same procedure, the tumor growth rate could not be estimated before biopsy. However, both cardiac CT and MRI demonstrated focal narrowing of the RIPV. After careful discussion with the patient regarding the potential risks and expected palliative benefits, pulmonary vein stenting was performed. Cardiac angiosarcoma could be considered a rare cause of pulmonary vein stenosis in patients presenting with unexplained dyspnea and a left atrial mass. Multimodality imaging combined with histopathologic confirmation is essential for diagnosis. In selected patients who are unsuitable for surgical treatment, ICE-guided pulmonary vein stenting can be performed safely and may provide effective palliation by restoring pulmonary venous flow and relieving symptoms.

## Data Availability

The raw data supporting the conclusions of this article will be made available by the authors, without undue reservation.
